# Objectivity of human consciousness is a product of tool usage

**DOI:** 10.3389/fpsyg.2014.01152

**Published:** 2014-10-09

**Authors:** Boris Kotchoubey

**Affiliations:** Institute of Medical Psychology and Behavioral Neurobiology, University of TübingenTübingen, Germany

**Keywords:** consciousness, goal-directedness, object, play, tool

Dijker (2014) has recently proposed a novel approach to consciousness, according to which the primary function of consciousness is producing *states of objectivity*. States of objectivity are defined (p.2) as “internally represent[ing] objects and their dispositional properties … in relatively stable, accurate, increasingly complete, perceiver-independent ways, unbiased by specific needs, motives, and anticipation of instrumental aspects and rewards.” I think that the author has made a very import point, and that the principal objectivity of (some kind of) conscious states is a major issue unfortunately missed in most contemporary theories of consciousness.

However, I also think that the publication of Dijker (2014) is only a first step, and that the view of consciousness as the capacity to produce states of objectivity needs further development. I hope that the following comments can contribute to this development.

First of all, I would argue that although Dijker' (2014) approach is original, it has predecessors. Particularly, this was what Franz Brentano (Brentano, 2008; 1st Ed. 1874) meant in his famous definition of consciousness as intentionality: each conscious state, according to Brentano, is “about” some object, which is outside consciousness. This object, therefore, does not belong to consciousness, and in this sense, it is objective.

Second, there are cases that quite obviously contradict the above definition of consciousness. Thus, pain experience is self-evidently not objective; therefore, according to the definition, it is not a conscious phenomenon. Such conclusion would have far-going practical consequences. It may imply, for instance, that having sufficiently strong muscle relaxants, surgeons do not need analgesics during their interventions. Whether surgical patients would be enthusiastic about this approach to consciousness, can be questioned.

However, this point is much less serious than it appears. The contradicting cases simply indicate that the theory should let down its level of generalization. Possibly, objectivity characterizes not all states of consciousness but only a particular class of such states, e.g., higher-order consciousness of Genaro (1996) and Rosenthal (2004), access consciousness of Block (2005, 2007), secondary consciousness of Edelman (1989), or cognitive consciousness of Panksepp (2005). The merits of Dijker's proposal will not be diminished by the fact that his ideas are related to an important subtype of conscious phenomena and not to each of them.

There is a more serious problem, however. The question remains open where the states of objectivity can come from. If I understand the author correctly, he believes that play behavior can result in conceiving of some aspects of the environment (e.g., children or sexual partner) as “fragile,” vulnerable and care-requesting. This hypothesis, however, does not clarify how the notions of vulnerability and care can help us to explain the phenomenon of objectivity. Relationships with vulnerable entities are emotional through and through. Everybody can only think on one's own small children, pets or just porcelain vessels. According to author's definition of states of objectivity, our interaction with these objects should be relatively stable, neutral, perceiver-independent, unbiased by our needs and motives. This is, of course, not the case.

Let us approach this issue from a different point. What is objective? Some aspects of Dijker' (2014) definition, such as accuracy and completeness, are difficult to test. Somebody must have standards of accuracy and completeness to determine in what degree my ideas of the world are accurate and complete. This being can be either God or other humans. In the former case the standards are absolute, but, unfortunately, we cannot know anything about them, because we are lacking divine knowledge. In the latter case we have to admit that also others' conscious states can be as little objective as ours, because other people have their own biasing factors. Thus, understood objectivity is nothing more than intersubjectivity. This is how the term “objectivity” is defined, e.g., in the test theory: a psychological test is objective if different test administrators and test evaluators come to the same conclusion. However, when Dijker speaks about objectivity and consciousness he obviously means much more than mere agreement between several (perhaps equally subjective) observers.

The matter does not get better if we suggest that objective features are such that “really exist” independently of any observer. This definition cannot be operationalized. How can we test that the feature F really exists if, by definition, such testing must not involve any observer?

On the other hand, other aspects of the definition can be more useful: independence of the perceiver's needs, motives and (I would add) his/her particular anatomical and physiological organization. The word “object” (whose derivate is “objectivity”) has quite similar etymology in virtually all European languages (Romanic, Germanic, and Slavonic). It means something that I have thrown in front of me, and now it stands there opposing to me. This, again, leads us back to Brentano who claimed that a mental state, in contrast to a physical state, necessarily contains (or “is directed toward”) something that is outside this state (its “intentional object”).

But how can I perform such an act of throwing outwards something (which, originally, belonged to me) in such a manner that it now appears against me, as a thing which is different from me and resists me? To do this operation, I need a tool.

The world as typically perceived by an animal is not an objective world in any sense of this word. Rather, it is an ecological world (“Lebenswelt”), variously described by biologists (e.g., von Uexküll, 1970), psychologists (Gibson, 1979), philosophers (Heidegger, 1963), and system theorists (Bruineberg and Rietveld, 2014). Unlike the world of adult humans, the ecological world does not contain objects, but constellations of properties immediately related to an organism. An apple is not an object; rather, it is something that I can grasp, take, eat, throw, etc. A horizontal surface is something where I can rest and sleep upon. A fence is an obstacle for my locomotion, which I can jump over or run around it. Everything is perceived as “affordance” (Gibson, 1979; Bruineberg and Rietveld, 2014), i.e., in immediate relation to my capabilities, my anatomic and physiological composition and my actual needs.

The situation becomes different as soon as I use a tool. A tool is an element of my environment that interacts not only with my organism, but also with other elements. The affordances of a banana (it can be grasped, eaten, etc.) are fully described in terms of its relation to the organism (because it is the organism who can grasp and eat it); but the properties of the stick used to reach the banana are also described in terms of its relation to the banana. For illustration, see Figure 1, in which the former properties are indicated as A, and the latter, as B. For example, the stick has to be hard and possibly sharp. We know from the famous experiments of Köhler (1926) that some apes tried to reach a banana using a bundle of hay, but they failed because hay lacks the necessary property of hardness. Thus, the organism, to use a tool, learns that some components of its environment possess properties which manifest themselves outside the relationships to this organism, i.e., objective properties. Given the relations of the A-type and those of the B-type (see Figure 1), the relations of the C-type emerge, i.e., the knowledge that *there is something in the world, which is not immediately related to me*.

**Figure 1:**
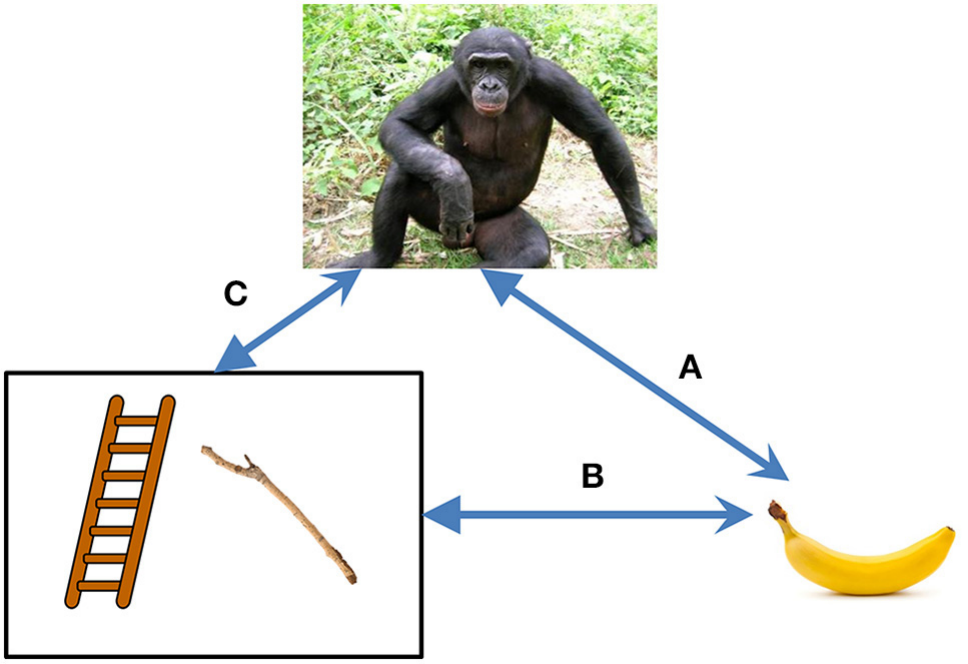
**Relationships between an organism (the ape), its goals (the banana), and tools (ladder, stock)**. Using the tools, the organism records the relationships of type B between the tool and the goal. Thereby, the organism gets the idea that some components in the environment are related, not only to the organism itself, but also to other components of the environment.

The hardness of the stick as a tool principally differs not only from the eatability of the apple but also from the hardness of a rock that I could collide with. It is important that the rock is hard, because this is why it may hurt me. It is important that the stick is hard because this is why it can move the banana. In the former case, the hardness manifests itself in relation to *me*; in the latter case, it manifests itself in relation to *something else*.

In other words: what happens if my organism is removed from the situation? If I am simply not here? There is no sense in such a situation to talk about the taste of an apple (who can taste it?) or the hardness of a rock (whom can it injury?), but the stick will remain hard, because its hardness follows from its interaction with another component of the environment. The stick, therefore, is an outside object whose properties (and, therefore, its very existence) do not depend on my presence or absence in the situation.

Now we can pass over to other aspects of objectivity, i.e., completeness and accuracy. How we can know that a property we perceive as objective (e.g., the hardness of the stick) is relatively complete and accurate? “By their fruit you will recognize them,” said Jesus (Matthew 7, 16). This fruit is the banana: if I was unable to reach it, this means that I failed to recognize real, objective properties of the employed tool. The banana, therefore, is the final criterion of objectivity: the fact that I can reach my goal using this tool proves that my perception of the tool's properties is sufficiently complete and accurate.

This is, however, an important critical point of my proposal: if, as I suggest, objective conscious states are derivate of tool usage, then our objectivity is only instrumental. It still remains in the space of the available experience.

This is true: my proposal implies that objectivity cannot be absolute. It cannot transcend the space between the organism and the goal of its activity. However, it can expand this space and even a simplest tool substantially broadens the organism's world view. A new, third component emerges between the organism and its goal, having properties unknown beforehand.

The space is further expanded when higher-order tools emerge. Whereas a first-order tool is a mean to reach a goal, a second-order tool is a mean to reach a first-order tool. Second-order tools can be used by crows (Taylor et al., 2007) and monkeys (Evans and Westergaard, 2006), while our ancestors such as Homo habilis probably possessed tools of very high orders (Ingold, 1993). When I do not just use an available element of the environment to reach another element (i.e., my goal), but rather, I use element A to make element B, which I need to make element C, and so forth, and the goal is only at the end of a very long chain of tools, then the instrumental background of objectivity can virtually disappear from awareness.

The presented view agrees with that of Dijker' (2014) at the point that the ultimate source of objectivity is play. Play is a necessary predecessor of tool usage (Huizinga, 1950). In order to use a component of the external world as a tool, an animal should first manipulate it without a particular aim, i.e., play with it. Moreover, there is a direct relationship between tool use, language, and intelligence (e.g., Gibson, 1993; Frey, 2008; Lefebvre, 2013). However, if—as suggested here—play is only a necessary but not sufficient stage in the development of tool usage, the latter can be expected to be less broadly spread in the animal world than the former. In fact, tool usage is limited to a few animal orders such as primates and corvidae, while play is observed among many mammals and birds including ungulates, carnivorans, parrots, and many others (Burghardt, 2005); there is even evidence of play in octopus (Kuba et al., 2006).

## Conflict of interest statement

The author declares that the research was conducted in the absence of any commercial or financial relationships that could be construed as a potential conflict of interest.
